# The Hospital Funds and the Special Hospitals

**Published:** 1904-12-17

**Authors:** 


### THE HOSPITAL FUNDS AND THE SPECIAL HOSPITALS.

We called the attention of the editor of the *Medical Press and Circular* to certain mis-statements in its issue of the 30th ult. which were obvious to us, realising that the article before publication must have escaped the editor's vigilant eye. We did this because we were confident that we were warranted in treating our contemporary as anxious to maintain on all occasions the honourable traditions of the Press by insisting upon accuracy of statement in matters of criticism. Our confidence has been fully justified in the result. The *Medical Press*, in its issue of the 14th instant, in the course of the first leading article states: "We have no intention of declining responsibility for the statement in question. The editor of The Hospital calls upon us in the name of responsible journalism to justify a certain phrase. In the name of responsible journalism, and in support of the honourable traditions that have always governed the *Medical Press and Circular*, we withdraw that phrase, and regret that it should have inadvertently crept into our columns."

We congratulate our contemporary upon the courageous and honourable course it has adopted in unreservedly withdrawing the statement, which action must increase the respect in which the *Medical Press and Circular* is deservedly held. The statements in question referred (1) to the action of the Sunday Fund which we disposed of last week; and (2) to "the host of small but deserving medical charities from which grants have been withheld by the Sunday and King Edward's Hospital Funds." The *Medical Press*, having now withdrawn the latter statement, and expressed regret that it should have inadvertently crept into its columns, there is nothing more to be said on the subject.

